# The Functional Significance of a Novel Conceptualization of Intrusion Symptoms of PTSD

**DOI:** 10.1192/j.eurpsy.2023.1020

**Published:** 2023-07-19

**Authors:** T. R. Spiller, G. Gross, O. Duek, R. H. Pietrzak, I. Harpaz-Rotem

**Affiliations:** 1 Psychiatric University Hospital Zurich, Zürich, Switzerland; 2 Department of Psychiatry, Yale University, New Haven, United States; 3 University of Zurich, Zurich, Switzerland; 4 National Center for PTSD; 5 Northeast Program Evaluation Center, VA Connecticut Healthcare System, West Haven, United States

## Abstract

**Introduction:**

Intrusion symptoms are a core defining feature of posttraumatic stress disorder (PTSD). It was recently proposed that intrusions may be comprised of two distinct underlying processes: internally-cued intrusions (e.g., memories), and externally-cued intrusions (e.g., reactions to one’s environment). Preliminary empirical evidence demonstrated superior fit of an 8-factor model of PTSD, separating intrusion symptoms into an in internally-cued and externally-cued symptom cluster over other factor models of PTSD. However, whether these two clusters are related differently with functional outcomes was not investigated previously.

**Objectives:**

This is the first study to examine the functional correlates of the internally-cued and externally-cued intrusion symptom clusters in PTSD to assess whether separating intrusion symptoms into these two clusters is of clinical and scientific relevance.

**Methods:**

Participants included 7460 veterans discharged from 40 VA PTSD residential treatment programs (RRTPs) across the United States in fiscal years 2018 through 2020. Demographic data was collected using a self-report form during the admission process. Symptoms of PTSD, anxiety, depression, and emotional and physical functioning were assessed with the PTSD Checklist for DSM-5, the Patient Health Questionnaire-9, the Generalized Anxiety Disorder Questionnaire-7, and the corresponding subscales of the Short Form 12-item Health Survey, respectively. Latent network modeling was used to test the fit of the 8-factor model of PTSD. Structural equation modelling was used to investigate the associations between the factors of PTSD and the functional outcomes. All associations were adjusted for demographic characteristics, and standardized.

**Results:**

The 8-factor model, with separate intrusion factors, showed good model fit (CFI 0.965, RMSEA 0.045, χ^2^ 2453.022, and P <.001). Internally-cued intrusions were negatively associated with physical functioning and positively with emotional functioning. No relationship with depression or anxiety was found. In contrast, externally-cued intrusions were negatively associated with emotional functioning and positively associated with anxiety, but not related to physical functioning and depression.

**Conclusions:**

This study provides initial support for the functional utility of distinguishing between internally- and externally-cued intrusions in veterans with PTSD. Consequently, researchers focusing on the biological underpinnings of intrusion symptoms (e.g., in imaging or genetic studies) should account for differences in the origin of the cue triggering intrusions. Our findings are of potential clinical relevance as they might help patients adapt their coping strategies for intrusions depending on whether they originate internally (e.g., thoughts) or externally (e.g., loud noises).

**Disclosure of Interest:**

None Declared

